# Intrinsic resistance and efficacy of immunotherapy in microsatellite instability-high colorectal cancer: A systematic review and meta-analysis

**DOI:** 10.17305/bjbms.2022.8286

**Published:** 2023-03-16

**Authors:** Ren Wang, Jie Lian, Xin Wang, Xiangyi Pang, Benjie Xu, Shuli Tang, Jiayue Shao, Haibo Lu

**Affiliations:** 1 Department of Outpatient Chemotherapy, Harbin Medical University Cancer Hospital, Harbin, China

**Keywords:** Colorectal cancer (CRC), microsatellite instability-high (MSI-H), intrinsic resistance, efficacy, immunotherapy

## Abstract

Some patients with microsatellite instability-high colorectal cancer (MSI-H CRC) have shown a poor response to immunotherapy in clinical trials. We investigated the intrinsic resistance to and efficacy of immunotherapy in patients with MSI-H CRC. The PubMed and Web of Science databases were searched using keywords such as “colorectal cancer,” “immunotherapy,” and “clinical experiment.” Random-effects models were used to generate the combined complete response, partial response, stable disease, progressive disease, objective response rate (ORR), disease control rate (DCR), and incidence of adverse events. We then performed a subgroup analysis based on the ORR and incidence of intrinsic resistance. The meta-analysis included seven clinical trials. The incidences of complete response, partial response, stable disease, and progressive disease summarized by the random-effects model were 8%, 37%, 26%, and 25%, respectively. The ORR and DCR were 45% and 71%, respectively. The ORRs of programmed cell death protein 1 inhibitor (anti-PD-1), programmed death ligand 1 inhibitor (anti-PD-L1), and anti-PD-1 combined with cytotoxic T lymphocyte-associated antigen 4 inhibitor (anti-CTLA-4) immunotherapy were 38%, 54%, and 57%, respectively. The ORR of immune checkpoint inhibitors for first- and third-line therapy was 56% and 32%, respectively. Dual-drug immunotherapy significantly reduced the incidence of intrinsic resistance to immunotherapy (12% vs 31%). The incidences of intrinsic resistance to first-line therapy and second-line and later therapy were 29% and 26%, respectively. Approximately 25% of patients with MSI-H CRC had intrinsic resistance to immunotherapy. Anti-PD-1 combined with anti-CTLA-4 significantly increased the ORR, thereby reducing the incidence of intrinsic resistance. Moving immunotherapy into earlier lines of therapy, although not reducing the incidence of intrinsic resistance, can improve the ORR in patients with MSI-H CRC.

## Introduction

Colorectal cancer (CRC) is the most common malignant cancer in the gastrointestinal tract. In 2020, there were more than 1.9 million new cases of CRC and 935,000 related deaths [1]. Statistical data show that the worldwide incidence of CRC is expected to increase to 2,500,000 by 2035 [2]. Systemic treatment of CRC has progressed in recent years; however, most patients with advanced disease lack effective treatment options after disease progression. Therefore, there is still an urgent need to explore new treatment modalities for patients with advanced CRC.

Immunotherapy has been proven to be an advanced and effective treatment for various solid tumors. Microsatellite instability (MSI) tumors have a higher tumor mutation burden and higher number of tumor-infiltrating lymphocytes, making them more suitable for immunotherapy [3]. The therapeutic efficacy of immune checkpoint inhibitors (ICIs) in patients with MSI-high (MSI-H) CRC is widely recognized. The National Comprehensive Cancer Network guidelines recommend pembrolizumab or nivolumab as second-line therapeutic options, especially for patients with MSI-H CRC. However, the objective response rate (ORR) of MSI-H CRC to immunotherapy is only 31.7%–62.5% in the clinical trials reported to date [4–10]. This means that some patients with CRC have intrinsic resistance to ICIs. In this study, we evaluated the intrinsic resistance, efficacy, and safety of immunotherapy for MSI-H CRC.

## Materials and methods

### Study design

This systematic review and meta-analysis followed the Preferred Reporting Items for Systematic Reviews and Meta-analyses (PRISMA). By applying the Problem/Population, Intervention, Comparison, and Outcome (PICO) framework, we defined the participants in this study. All participants received immunotherapy. In all included studies, the “Comparison” element of the PICO framework was not involved because we performed a pooling of single-group rates. The primary outcomes were complete response (CR), partial response (PR), stable disease (SD), progressive disease (PD), ORR, and disease control rate (DCR).

### Search strategy

We conducted a comprehensive search of the PubMed and Web of Science online databases to identify clinical trials on immunotherapy for MSI-H CRC in April 2022. The search terms were as follows: “colorectal cancer” or “colon cancer” or “rectal cancer” and “immunotherapy” or “programmed cell death protein 1 inhibitor” or “programmed death ligand 1 inhibitor” or “cytotoxic T lymphocyte-associated antigen 4 inhibitor” and “clinical trials.” To identify other relevant studies, the reference lists of eligible studies and similar literature were manually searched. We also searched the clinicaltrials.gov registry for eligible clinical trials.

### Selection of studies

Two reviewers independently performed a preliminary screening of the titles and abstracts after excluding duplicates. The inclusion criteria for eligible studies were involvement of patients with MSI-H CRC, prospective clinical trial, administration of immunotherapy, and provision of detailed real-world outcome data on treatment efficacy. Disagreements between the two reviewers were resolved by discussion with a third person.

### Data extraction

The primary outcomes were CR, PR, SD, PD, ORR, and DCR. The secondary outcomes were 1-year overall survival (OS), 1-year progression-free survival (PFS), and the incidence of grade ≥ 3 adverse events (AEs). The best observed response (BOR) is an important criterion for measuring the antitumor effect of an experimental drug. According to the Response Evaluation Criteria in Solid Tumors (RECIST) version 1.1, the BOR was defined as the best efficacy recorded from the beginning of treatment until disease progression or recurrence. The ORR was defined as the proportion of patients with CR and PR. The DCR was defined as the proportion of patients with CR, PR, and SD. The grades of AEs were determined according to the Common Terminology Criteria for Adverse Events (CTCAE).

Data were extracted and collected independently by two individuals. The extracted information included the corresponding author, country, year of publication, the number of patients, phase, sample size, disease stage, type of immunosuppressant, CR, PR, SD, PD, ORR, DCR, 1-year OS, and 1-year PFS. We directly extracted the raw data of the article and calculated part of the data from the original data.

### Ethical statement

Ethics approval was not needed because of the nature of this study (meta-analysis).

### Statistical analysis

The *I^2^* test was used to estimate study heterogeneity. We chose a random-effects model in advance considering the high heterogeneity of the data. First, we summarized CR, PR, SD, PD, ORR, DCR, 1-year OS, 1-year PFS, and incidence of grade ≥ 3 AEs. Next, a subgroup analysis of ICI types and immunotherapy lines was performed based on the ORR and the incidence of intrinsic resistance. We then summarized the ORRs for *BRAF*-mutant and *BRAF*-wild patients. The *χ^2^* test was used to compare the differences in ORR between dual-drug immunotherapy and single-drug immunotherapy in different *BRAF* states. In addition, a sensitivity analysis was performed to explore the robustness of the results and potential sources of heterogeneity. Egger’s bias test and a funnel plot were used to evaluate the risk of publication bias in the included studies. All analyses were performed by STATA version 13 (StataCorp, College Station, TX, USA) and SPSS Version 18 (SPSS Inc., Chicago, IL, USA).

**Figure 1.**
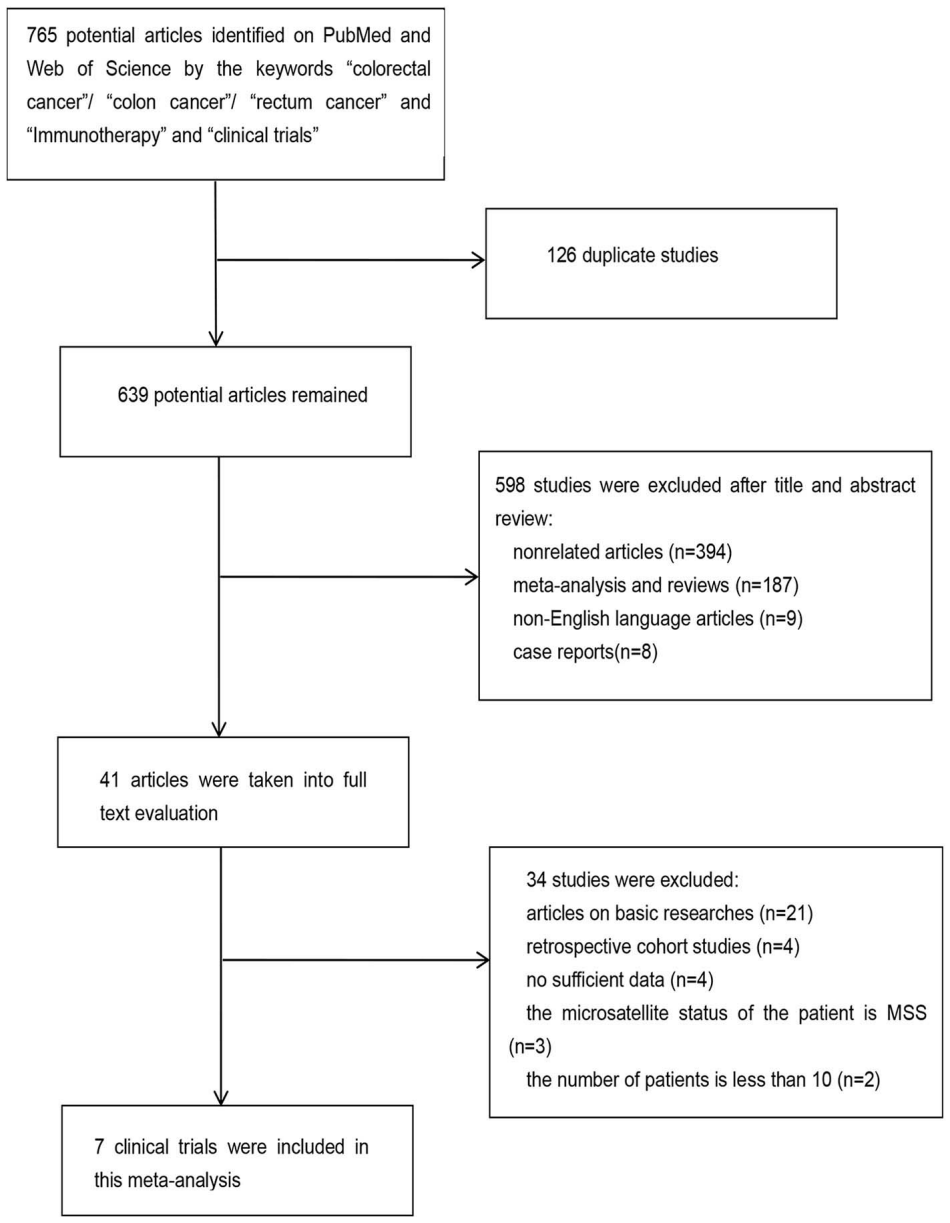
**Flowchart of search and study selection**. MSS: Microsatellite-stable.

## Results

### Included studies

This meta-analysis included 7 clinical trials involving 604 patients with MSI-H CRC [4–10]. The search identified 765 articles, of which 126 duplicates were excluded. Another 598 articles were excluded based on evaluation of the title and abstract (394 articles did not refer to patients with MSI-H CRC, 187 were meta-analyses and reviews, 9 were not published in English, and 8 were case reports). Another 41 articles were excluded after a full-text review (including 2 studies involving < 10 cases). Two clinical trials were excluded because their primary outcome was the tumor regression grade. Finally, this meta-analysis included seven clinical trials of ICIs in patients with MSI-H CRC. CheckMate 142, a phase II study in patients with MSI-H CRC, was conducted in two parts. These two parts of the study screened patients separately and reported the efficacy of nivolumab monotherapy and nivolumab combined with ipilimumab [9, 10]. Figure 1 illustrates the process of the literature screening. Of the seven clinical trials, four were conducted in the United States, one in China, one in South Korea, and one in France. Only one study was a phase III clinical trial; the remaining six were phase II clinical trials. In seven clinical trials, the type of ICIs used were as follows: programmed cell death protein 1 inhibitor (anti-PD-1) in three studies, a programmed death ligand 1 inhibitor (anti-PD-L1) in two studies, and anti-PD-1 combined with cytotoxic T lymphocyte-associated antigen 4 inhibitor (anti-CTLA-4) in two studies. Table 1 summarizes the features of the included studies.

### Efficacy

**Figure 2.**
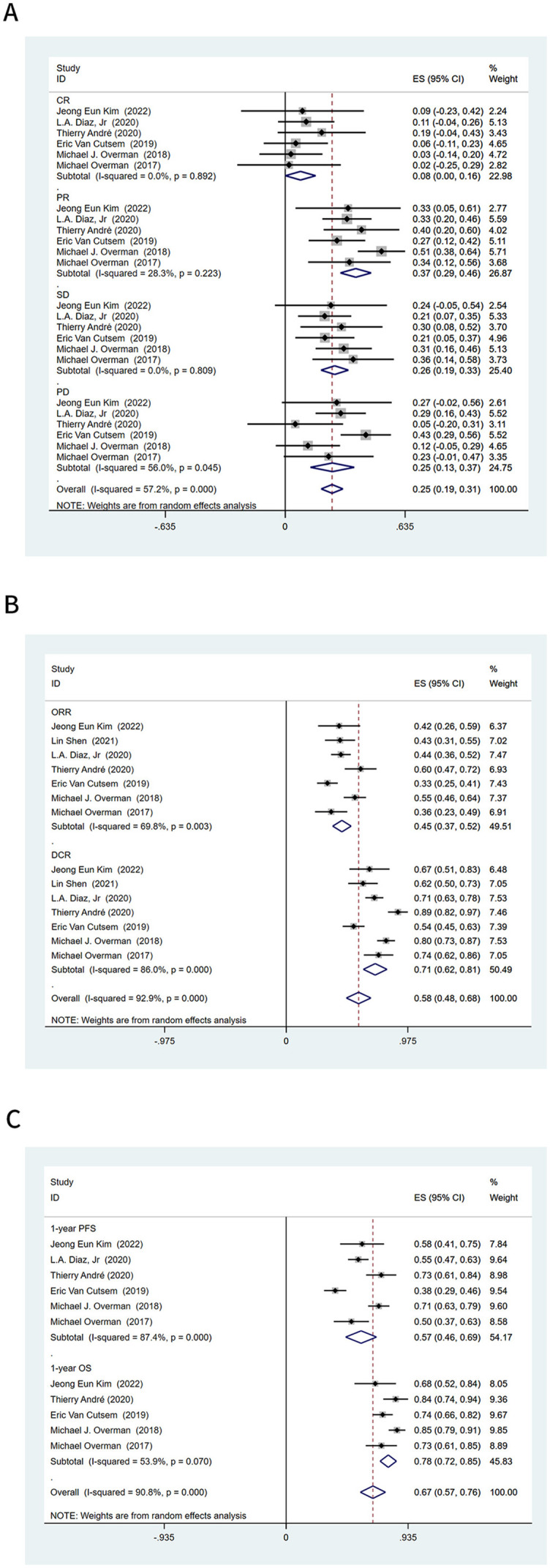
**Efficacy of immunotherapy in patients with microsatellite instability-high colorectal cancer.** (A) Pooled CR, PR, SD, and PD; (B) Pooled ORR and DCR; (C) Pooled 1-year PFS rate and 1-year OS rate. ES: Effect size; CI: Confidence interval; CR: Complete response; PR: Partial response; SD: Stable disease; PD: Progressive disease; ORR: Objective response rate; DCR: Disease control rate; PFS: Progression-free survival; OS: Overall survival.

Six studies recorded the BOR in immunotherapy. The pooled CR was 8% (95% confidence interval [CI] 0.00–0.16), PR was 37% (95% CI 0.29–0.46), SD was 26% (95% CI 0.19–0.33), and PD was 25% (95% CI 0.13–0.37) (Figure 2A).

**Figure 3.**
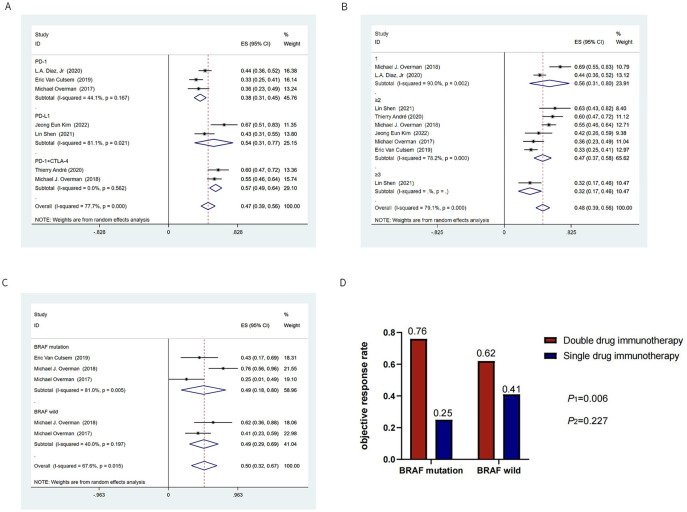
**Subgroup analysis based on objective response rate.** (A) Subgroup analysis of immune checkpoint inhibitors; (B) Subgroup analysis of lines of immunotherapy; (C) Subgroup analysis of *BRAF* status; (D) Histogram of single- or dual-drug immunotherapy and *BRAF* mutation status. * *χ2* test was used to calculate *P1* for ORR in patients with *BRAF*-mutant receiving dual-drug immunotherapy or single-drug immunotherapy; *χ2* test was used to calculate *P2* for ORR in patients with *BRAF*-wild receiving dual-drug immunotherapy or single-drug immunotherapy. ES: Effect size; CI: Confidence interval; PD-1: Programmed cell death protein 1; PD-L1: Programmed death ligand 1; CTLA-4: Cytotoxic T lymphocyte-associated antigen 4; ORR: Objective response rate.

**Figure S1.**
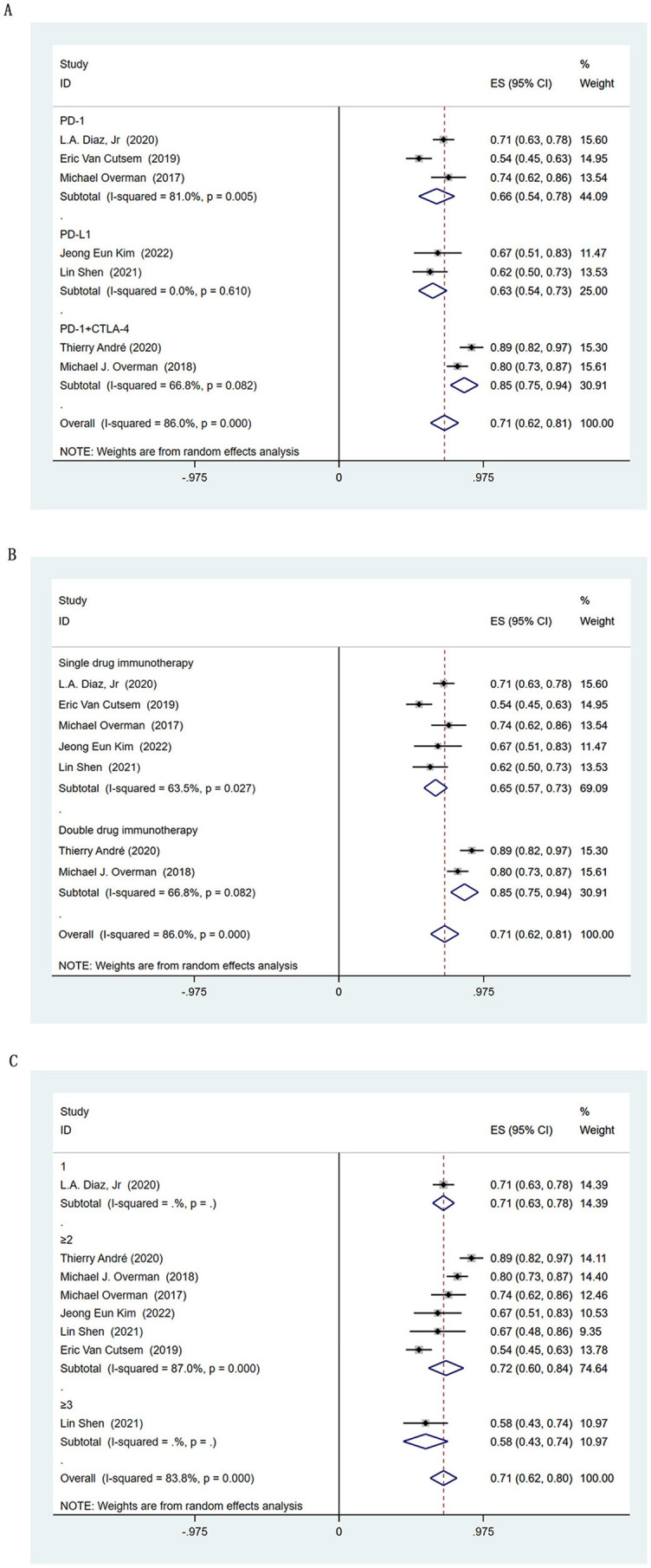
**Subgroup analysis based on disease control rate.** (A) Subgroup analysis of immune checkpoint inhibitor (anti-PD-1, anti-PD-L1, anti-PD-1 + anti-CTLA-4); (B) Subgroup analysis of single- and dual-drug immunotherapy; (C) Subgroup analysis of lines of immunotherapy. PD-1: Programmed cell death protein 1; PD-L1: Programmed death ligand 1; CTLA-4: Cytotoxic T lymphocyte-associated antigen 4.

The ORR and DCR were provided in all seven studies. The ORR of all 604 patients with MSI-H CRC was 45% (95% CI 0.37–0.52), and the DCR was 71% (95% CI 0.62–0.81) (Figure 2B). The pooled 1-year PFS was 57% (95% CI 0.46–0.69) and 1-year OS was 78% (95% CI 0.72–0.85) (Figure 2C). In the subgroup analysis based on the type of ICIs, anti-PD-L1 had a higher ORR than anti-PD-1 (54% vs 38%, respectively), and anti-PD-1 combined with anti-CTLA-4 had the highest ORR of 57% (95% CI 0.49–0.64) (Figure 3A). In the subgroup analysis based on the type of ICIs, the DCR for anti-PD-1, anti-PD-L1, and anti-PD-1 combined anti-CTLA-4 immunotherapy were 66%, 63%, and 85%, respectively (Figure S1A). Further pooling analysis found that the DCR of single-drug immunotherapy and dual-drug immunotherapy were 65% and 85%, respectively (Figure S1B). This emphasizes the advantage of dual-drug immunotherapy. When exploring the application opportunity of immunotherapy, we found that patients who received ICIs as first-line therapy achieved the highest ORR, reaching 56% (95% CI 0.31–0.80). Patients receiving ICIs as third-line therapy achieved the lowest ORR, only 32% (95% CI 0.17–0.46) (Figure 3B). This suggests that ICIs may achieve better results in the early treatment of advanced MSI-H CRC. However, there was no clear trend change in DCR-based subgroup analyses (Figure S1C).

**Table 1:** Characteristics of included studies

**Study**	**Country**	**Design**	**Clinical number**	**Phase**	**Sample size**	**ICIs**	**Lines of immunotherapy**	**Target**	**Resistance**	**ORR**	**DCR**	**1-year OS rate**	**1-year PFS rate**	**Incidence of grade 3-4 AEs**
Jeong Eun Kim, 2022	Korea	RCT	NCT03435107	II	33	Durvalumab	≥ 2	PD-L1	27.3%	42.4%	66.7%	68.3%	58.2%	36.4%
Lin Shen, 2021	China	RCT	NCT03667170	II	24	Envafolimab	2	PD-L1	–	62.5%	66.7%	–	–	–
					41	Envafolimab	≥ 3	PD-L1	–	31.7%	58.5%	–	–	–
L.A. Diaz, Jr, 2020	U.S.A	RCT	NCT02563002	III	153	Pembrolizumab	1	PD-1	29.4%	43.8%	70.6%	-	55.3%	56.0%
Thierry André, 2020	French	RCT	NCT03350126	II	57	Nivolumab + ipilimumab	≥ 2	PD-1+CTLA-4	–	59.6%	89.5%	84.0%	72.9%	29.8%
Eric Van Cutsem, 2019	U.S.A	RCT	NCT02460198	II	124	Pembrolizumab	≥ 2	PD-1	43.0%	33.0%	54.0%	74.0%	37.6%	14.5%
Michael J. Overman, 2018	U.S.A	RCT	NCT02060188	II	119	Nivolumab + ipilimumab	≥ 2	PD-1+CTLA-4	12.0%	55.0%	80.0%	85.0%	71.0%	32.0%
Michael Overman, 2017*	U.S.A	RCT	NCT02060188	II	53	Nivolumab	≥ 2	PD-1	21.0%	36.0%	74.0%	73.0%	50.0%	20.0%

*CheckMate 142 is the phase 2 study performed to assess the activity and safety of nivolumab monotherapy or nivolumab in combination with ipilimumab in patients with microsatellite instability-high colorectal cancer. ICIs: Immune checkpoint inhibitors; Resistance: Rate of intrinsic resistance during immunotherapy; ORR: Objective response rate; DCR: Disease control rate; OS: Overall survival; PFS: Progression-free survival; AEs: Adverse events; RCT: Randomized controlled trial; CTLA-4: Cytotoxic T lymphocyte-associated antigen 4; PD-1: Programmed cell death protein 1; PD-L1: Programmed death ligand 1.

*BRAF* is an important gene for CRC, and *BRAF*-mutant CRC accounts for about 10%–15% of MSI-H CRC. The ORR for *BRAF*-mutant patients was available from 3 studies involving 43 patients. In the subgroup analysis, *BRAF*-mutant and *BRAF*-wild patients achieved the same ORR (49%) after immunotherapy (Figure 3C). This suggests that *BRAF* mutation is not a predictive biomarker of the response to immunotherapy in patients with MSI-H CRC. Our results also showed that patients with *BRAF*-mutant CRC who received dual-drug immunotherapy achieved a better ORR than those who received single-drug immunotherapy (*P1* ═ 0.006). However, in *BRAF*-wild CRC, there was no statistically significant difference in ORR between those receiving dual-drug immunotherapy and single-drug immunotherapy (*P2* ═ 0.227), despite a trend toward better efficacy (Figure 3D).

### Intrinsic resistance rate

A total of 25% MSI-H CRC patients had PD as BOR after receiving immunotherapy (95% CI 0.13–0.37) (Figure 2A), suggesting that some patients with MSI-H CRC may have intrinsic resistance to ICIs. This means that 25% of patients with MSI-H CRC do not benefit from immunotherapy.

**Figure 4.**
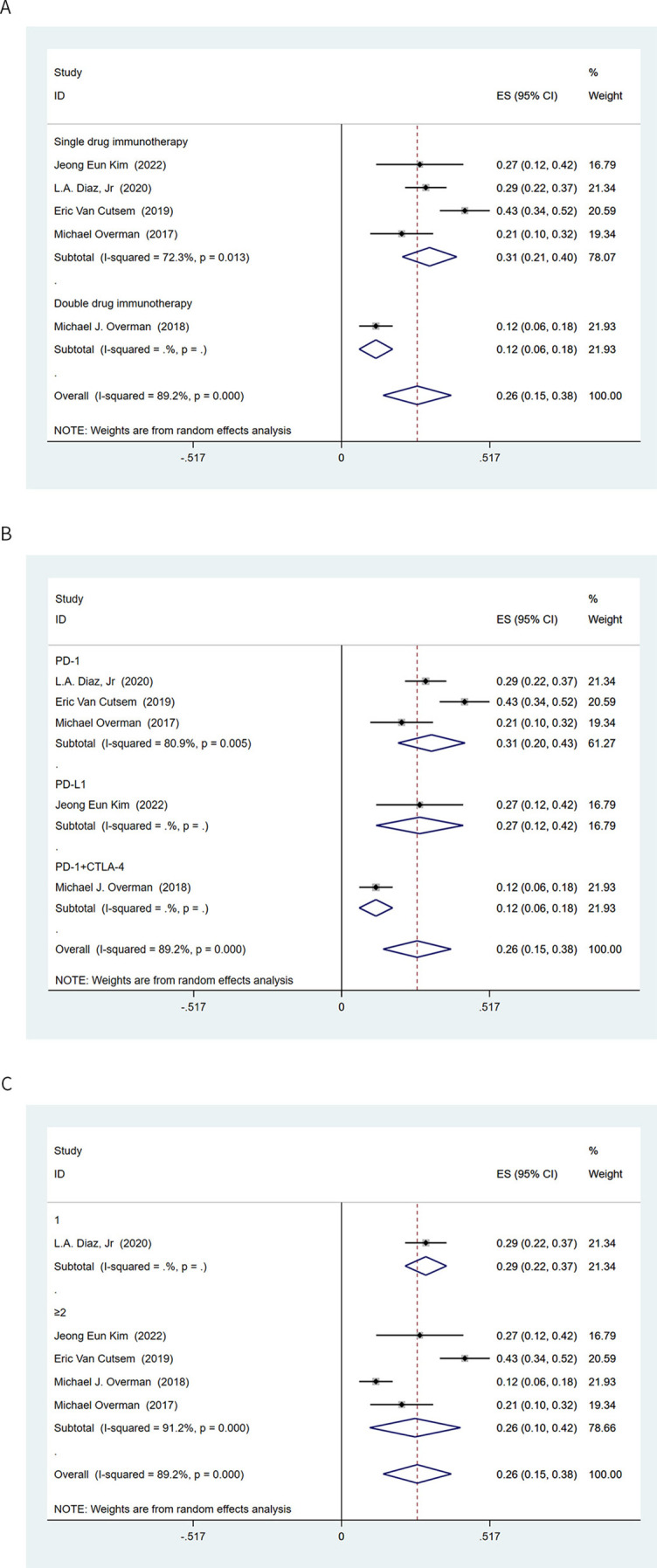
**Subgroup analysis based on rate of intrinsic resistance**. (A) Subgroup analysis of single- and dual-drug immunotherapy; (B) Subgroup analysis of immune checkpoint inhibitors; (C) Subgroup analysis of lines of immunotherapy. ES: Effect size; CI: Confidence interval; PD-1: Programmed cell death protein 1; PD-L1: Programmed death ligand 1; CTLA-4: Cytotoxic T lymphocyte-associated antigen 4.

The incidence of intrinsic resistance to single-drug immunotherapy was 31% (95% CI 0.21–0.40), and further analysis showed that the rate of intrinsic resistance to dual-drug immunotherapy was 12% (95% CI 0.06–0.18) (Figure 4A). The incidence of intrinsic resistance to anti-PD-1 and anti-PD-L1 in patients with MSI-H CRC was 31% (95% CI 0.20–0.43) and 27% (95% CI 0.12–0.42), respectively (Figure 4B). When exploring the application opportunity of immunotherapy, we found that, although early immunotherapy can improve the ORR (Figure 3B), it did not significantly change the incidence of intrinsic resistance (first-line vs second- or later-line treatment: 29% vs 26%, respectively) (Figure 4C).

### Safety

**Figure S2.**
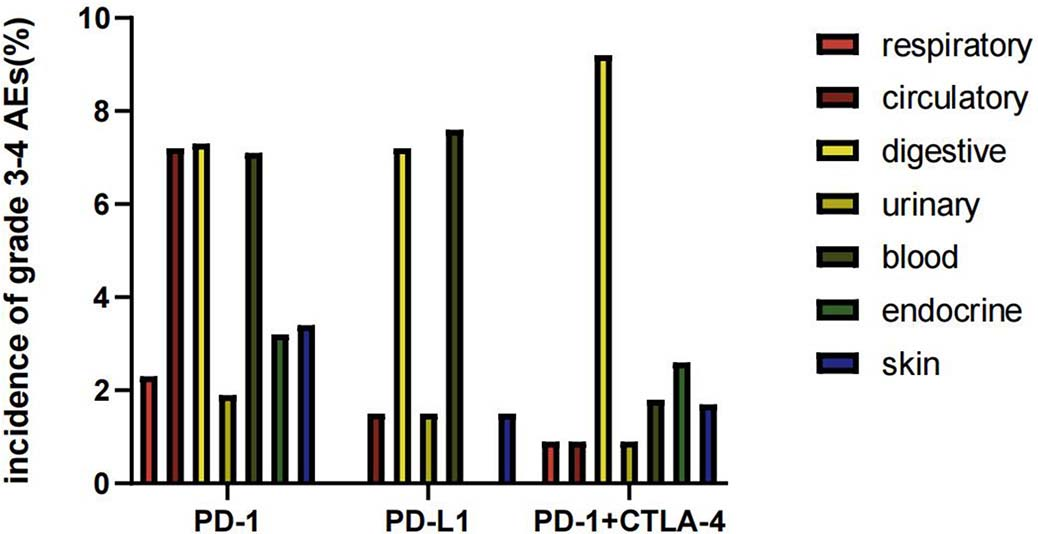
**Incidence of grade 3-4 adverse events (%) in each system according to immune checkpoint inhibitors (anti-PD-1, anti-PD-L1, anti-PD-1 + anti-CTLA-4).** PD-1: Programmed cell death protein 1; PD-L1: Programmed death ligand 1; CTLA-4: Cytotoxic T lymphocyte-associated antigen 4; AEs: Adverse events.

We counted the number of grade ≥ 3 AEs among the clinical trials. ICIs can cause damage to the respiratory, circulatory, urinary, hematologic, and other systems. Our results showed that the AEs of immunotherapy mainly involved the digestive and hematologic systems (Figure S2). The most common AEs were increased lipase concentration, liver function impairment, and anemia. The incidence of grade ≥ 3 of those AEs were 9.1%, 4.4%, and 5.2%, respectively (Figure S2).

### Publication bias and evaluation of reference quality

**Figure 5.**
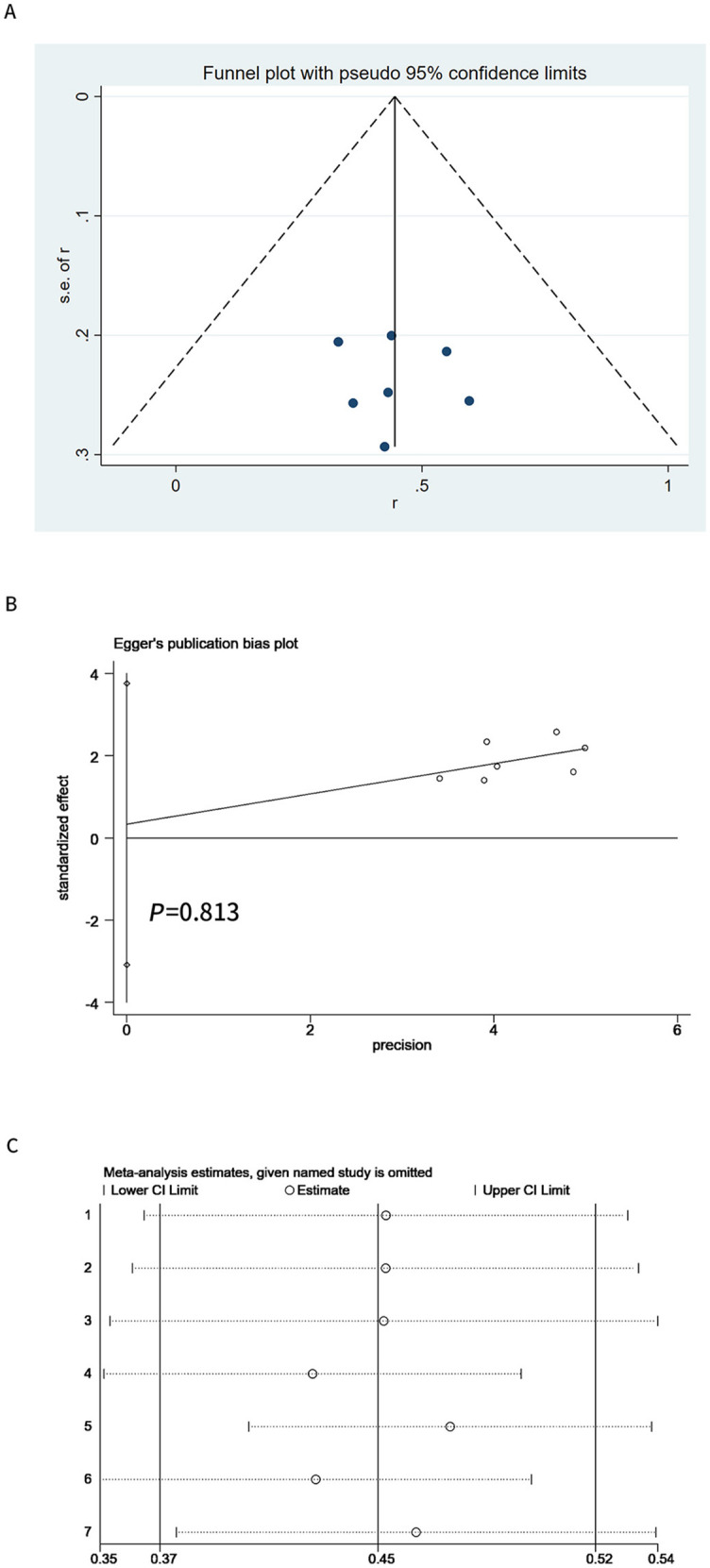
**Funnel plot and Egger’s plot.** (A) Funnel plot based on objective response rate; (B) Egger’s publication bias plot based on objective response rate; (C) Sensitivity analysis based on objective response rate. Every horizontal line represents the combined objective response rate and the 95% confidence interval after omitting the included studies one by one. CI: Confidence interval.

**Figure S3.**
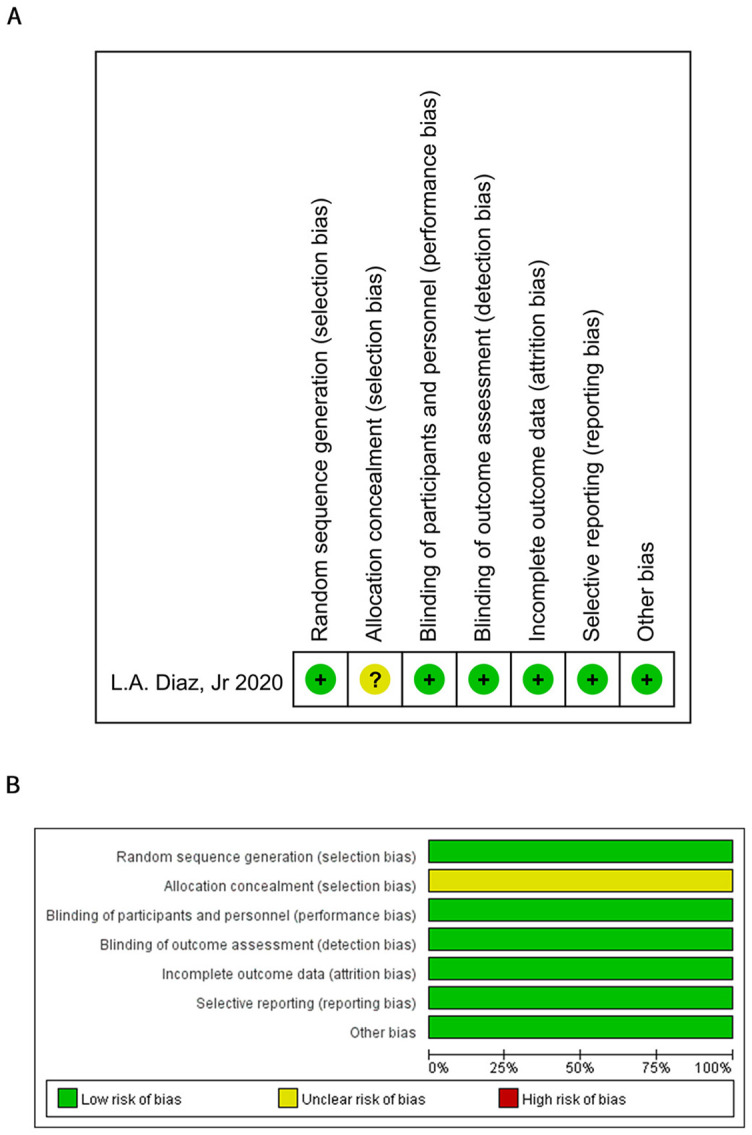
Results of literature quality evaluation in randomized controlled trials with the Cochrane risk-of-bias tool.

Funnel plot (Figure 5A) and the Egger test (Figure 5B) proved that publication bias of the included studies did not exist (*P* ═ 0.508). One randomized controlled study assessed literature quality using the Cochrane Risk of bias tool (Figure S3). The remaining six studies assessed literature quality using the MINORS methodological measure. All included studies scored above 10 points (Table S1).

## Discussion

This study is the first meta-analysis to definitively focus on the efficacy and safety of ICI therapy in patients with MSI-H CRC. In particular, this study also explored primary resistance to MSI-H CRC immunotherapy.

Our results showed that the pooled ORR was 45% (95% CI 0.37–0.52) and that the DCR was 71% (95% CI,0.62–0.81). Although ICIs have unique efficacy and significant survival benefits for MSI-H CRC patients, our results demonstrated that immunotherapy in patients with MSI-H CRC has not achieved the expected high efficacy. We performed a subgroup analysis to explore the factors affecting the efficacy of immunotherapy to better guide clinical practice. In this study, dual-drug immunotherapy had a more significant ORR than single anti-PD-L1 or anti-PD-1 immunotherapy (38% vs 54% vs 57%, respectively). CTLA-4 and PD-1/PD-L1 are two T cell inhibitory receptors with independent mechanisms of action. The combination of anti-PD-1/PD-L1 and anti-CTLA-4 increases effector T cell infiltration. The synergistic effect of the two enhances the antitumor immune response in different T cells and different microenvironments [11, 12]. Therefore, dual-drug immunotherapy can provide a higher ORR and satisfactory survival rate. It is a better treatment option for patients with advanced MSI-H CRC.

Not just dual-drug immunotherapy but also other treatment modalities in combination with immunotherapy appear to improve treatment efficacy. Targeted drugs, which inhibit tumor growth without adversely affecting the immune system, are potential treatment options in combination with ICIs. Moreover, studies have shown that radiation generated during radiotherapy can upregulate the intratumoral T cell chemokines CXCR3 and CCR5, driving the infiltration of antigen-specific CD8+ T cells [13]. Therefore, radiotherapy combined with ICIs may have synergistic antitumor effects. Unfortunately, most of the current clinical trials of immunotherapy for CRC are focused on enhancing the sensitivity of immunotherapy for patients with microsatellite-stable tumors. Several ongoing clinical trials are investigating the efficacy and safety of immunotherapy combined with targeted therapy or chemotherapy in patients with MSI-H CRC, but no clear results have been obtained (NCT04715633, NCT04301557, and NCT05035381). Our results showed that earlier immunotherapy was associated with a higher ORR. Clinical trials have confirmed that neoadjuvant immunotherapy can produce stronger and broader immune response. Neoadjuvant immunotherapy can achieve PR in 95% of patients with MSI-H CRC and CR in 60% of patients [14]. In one clinical trial of CRC, the ORR of 41 patients who received third-line immunotherapy was 31.7%, while that of 24 patients who received second-line immunotherapy was 62.5% [5].

Immunotherapy uses the body’s own immune system to kill the tumor cells. At present, there are two theories about the mechanism of immunotherapy-related AEs: generalized immune activation and the production of proinflammatory cytokines [15]. PD-1/PD-L1 is an immunonegative regulatory molecule; anti-PD-1/anti-PD-L1 can reverse the inhibition of downregulation of T cell activity, while anti-CTLA-4 can restore the T cell activation signal. Unlike precisely targeted therapies, immunotherapy results in widespread immune system activation that may affect multiple organs in the body [16]. In addition, a basic experiment showed that both anti-CTLA-4 and anti-PD-1/anti-PD-L1 led to proinflammatory cytokine aggregation. Anti-CTLA-4 induces more memory Th17 inflammatory cells than anti-PD-1/anti-PD-L1, which makes it more likely to lead to recurrence of immune-related AEs [15]. However, in the present study, the patients showed high tolerance to anti-PD-L1, anti-PD-1, or PD-L1 combined with CTLA-4 immunotherapy, and there was no clear difference in the incidence of AEs across organs.

ICIs are an efficient and safe treatment option for patients with MSI-H CRC. However, patients with MSI-H CRC exhibiting resistance to immunotherapy have been frequently reported in the last two years [17, 18]. A lack of response to ICIs was also seen in some of the patients in our included clinical trials. These patients had intrinsic resistance to immunotherapy. We have explored the reasons for the occurrence of intrinsic resistance to immunotherapy. Tumor cells evolve to select specific mechanisms for evading immune recognition. One of the most common mechanisms of MSI CRC immune evasion are mutations in beta-2-microglobulin. These mutations affect the expression of class I major histocompatibility complexes, thereby reducing the tumor cell response to PD-1/anti-PD-L1 [19]. In parallel, we should focus on the impact of the tumor microenvironment on immunotherapy. The presence of infiltrating lymphocytes in tumor tissue appears to be essential for ICIs to function [20]. Although MSI tumors have more T cell infiltration than microsatellite-stable tumors, there is also a relative lack of some antigen-reactive T cells in MSI tumors, which may be related to the immune resistance of patients with MSI-H CRC. Anti-PD-1 and anti-CTLA-4 initiate antitumor immunity by affecting different types of T cells [21]. These two therapies can withstand the resistance to immunotherapy through complementary mechanisms. In our study, a lower incidence of intrinsic resistance was also observed with dual-drug immunotherapy. In addition to genetic alterations and immune resistance caused by the tumor microenvironment, there is also a correlation between tumor metabolism and the immune response. A study investigating MSI-H CRC tumor metabolism showed that hypoxic tumors may produce too much lactic acid during glycolysis, thereby negatively regulating CD8 T cells [22]. Notably, false-positive MSI-H results may also have an impact. One clinical trial referred to the discrepancy between polymerase chain reaction results and immunohistochemistry results. Four patients were diagnosed with mismatch repair deficiency by immunohistochemistry but were proved to have microsatellite-stable tumors based on polymerase chain reaction [4]. There is no way to change resistance due to genetic mutations or the tumor’s own immune evasion mechanisms, but efforts should be made to circumvent the failures caused by testing.

In fact, the search for effective biomarkers remains a major task in immunotherapy and can help to screen populations that can benefit from immunotherapy. Our study showed that MSI-H may not be a perfect biomarker for predicting the efficacy of immunotherapy and that efforts should be made to explore better biomarkers for predicting efficacy. Several biomarkers that have been agreed upon to predict the efficacy of ICIs include the tumor mutation burden and PD-L1 expression level. Patients with a high tumor mutation burden also have an increased frequency of mutations, which increases the likelihood that the immune system will recognize immunogenic tumor neoantigens [23]. PD-L1 expression, as a direct target of PD-1 monoclonal antibody, is an important marker in tumor tissues and the marker that has undergone the most in-depth research. However, many factors influence PD-L1 expression, and the impact of PD-L1 expression on efficacy in different parts of the body is also different [24]. Furthermore, some epigenetic markers are considered more accurate biomarkers of the immunotherapy response. Recent evidence suggests that 5-methylcytosine is essential for regulating T cell proliferation and maintaining cytotoxicity and the differentiation of helper T cells; thus, 5-methylcytosine may serve as a prognostic and predictive biomarker for ICIs [25]. However, we still need to consider the cost and feasibility of this more precise epigenetic marker as a potential biomarker of the cancer immunotherapy response.

We included seven clinical studies summarizing the intrinsic resistance and efficacy of immunotherapy for MSI-H CRC. Because of the lack of primary data, only intrinsic resistance to immunotherapy was explored to some extent, and the clinicopathological characteristics of resistant patients could not be explored in depth. Furthermore, there is a lack of data from clinical studies of acquired resistance. Therefore, more research is still needed to gain insight into the specific characteristics of resistant patients and to properly guide clinical immunotherapy.

## Conclusion

A total of 25% of patients with MSI-H CRC have intrinsic resistance to immunotherapy. Anti-PD-1 combined with anti-CTLA-4 significantly increased the ORR in patients with MSI-H CRC, thereby reducing the incidence of intrinsic resistance. Moving immunotherapy into earlier lines of therapy, although not reducing the incidence of intrinsic resistance, can improve the ORR in patients with MSI-H CRC.

## Supplemental Data

**Table S1:** Results of literature quality evaluation with the methodological index for non-randomized studies (minors)

**Study**	**General standards**	**Additional standards**	**Total score**
Jeong Eun Kim, 2022	9	2	11
Lin Shen, 2021	12	2	14
Thierry André, 2020	8	2	10
Eric Van Cutsem, 2019	12	8	20
Michael J. Overman, 2018	11	2	13
Michael Overman, 2017	10	2	12
